# A continuous quality improvement strategy to strengthen screening practices and facilitate the routine use of intravenous iron for treating anaemia in pregnant and postpartum women in Nigeria: a study protocol

**DOI:** 10.1186/s43058-023-00400-y

**Published:** 2023-03-07

**Authors:** Ejemai Eboreime, Aduragbemi Banke-Thomas, Chisom Obi-Jeff, Yusuf Adelabu, Mobolanle Balogun, Adejoke A. Aiyenigba, Esther O. Oluwole, Opeyemi R. Akinajo, Bosede B. Afolabi

**Affiliations:** 1 Department of Psychiatry, Faculty of Medicine and Dentistry, 1E1 Walter Mackenzie Health Sciences Centre (WMC), University of Alberta, 8440 112 St NW, Edmonton, AB T6G 2B7 Canada; 2 Global Maternal and Newborn Health Hub, Institute of Lifecourse Development, University of Greenwich, London, UK; 3 Faculty of Epidemiology and Population Health, London School of Hygiene and Tropical Medicine, London, UK; 4 Brooks Insights Limited, Abuja, FCT Nigeria; 5 Department of Medicine, Faculty of Clinical Sciences, College of Medicine, University of Lagos, Lagos, Nigeria; 6 Department of Community Health & Primary Care, Faculty of Clinical Sciences, College of Medicine, University of Lagos, Lagos, Nigeria; 7 Department of Family Medicine, Korlebu Teaching Hospital, Accra, Ghana; 8 Department of Obstetrics and Gynaecology, Lagos University Teaching Hospital, Idi-Araba, Lagos, Nigeria; 9 Department of Obstetrics & Gynaecology, Faculty of Clinical Sciences, College of Medicine, University of Lagos, Lagos, Nigeria

**Keywords:** Iron deficiency anaemia, Pregnant women, Postpartum period, Parenteral iron, Continuous quality improvement, Ferric carboxymaltose, Nigeria

## Abstract

**Background:**

Pregnancy-related anaemia is a public health challenge across Africa. Over 50% of pregnant women in Africa get diagnosed with this condition, and up to 75% of these are caused by iron deficiency. The condition is a significant contributor to the high maternal deaths across the continent and, in particular, Nigeria, which accounts for about 34% of global maternal deaths. Whereas oral iron is the mainstay treatment for pregnancy-related anaemia in Nigeria, this treatment is not very effective given the slow absorption of the medication, and its gastrointestinal adverse effects which lead to poor compliance by women. Intravenous iron is an alternative therapy which can rapidly replenish iron stores, but fears of anaphylactic reactions, as well as several misconceptions, have inhibited its routine use. Newer and safer intravenous iron formulations, such as ferric carboxymaltose, present an opportunity to overcome some concerns relating to adherence. Routine use of this formulation will, however, require addressing misconceptions and systemic barriers to adoption in the continuum of care of obstetric women from screening to treatment. This study aims to test the options to strengthen routine screening for anaemia during and immediately after pregnancy, as well as evaluate and improve conditions necessary to deliver ferric carboxymaltose to pregnant and postpartum women with moderate to severe anaemia.

**Methods:**

This study will be conducted in a cluster of six health facilities in Lagos State, Nigeria. The study will employ continuous quality improvement through the Diagnose-Intervene-Verify-Adjust framework and Tanahashi’s model for health system evaluation to identify and improve systemic bottlenecks to the adoption and implementation of the intervention. Participatory Action Research will be employed to engage health system actors, health services users, and other stakeholders to facilitate change. Evaluation will be guided by the consolidated framework for implementation research and the normalisation process theory.

**Discussion:**

We expect the study to evolve transferable knowledge on barriers and facilitators to the routine use of intravenous iron that will inform scale-up across Nigeria, as well as the adoption of the intervention and strategies in other countries across Africa.

**Supplementary Information:**

The online version contains supplementary material available at 10.1186/s43058-023-00400-y.

Contributions to the literature

- This implementation research is expected to contribute to both contextual and generalisable knowledge on how to adopt and scale up the use of intravenous ferric carboxymaltose for treating obstetric cases of iron deficiency anaemia in Africa.
- Incorporating the Tanahashi health systems model into the Diagnose-Intervene-Verify-Adjust (DIVA) model, an adaptation of the Deming cycle, will demonstrate the application of systems thinking in quality improvement in resource-constrained health system contexts.
- Lessons learned from the methodological approach used in this study are expected to build capacity and advance the application of implementation science to strengthen health systems in Africa and beyond.

## Background

Anaemia in pregnancy (haemoglobin (Hb) levels less than 11.0g/dL) is a major public health burden with a higher incidence in low- and middle-income countries (LMICs) [1]. An estimated 31–52% of pregnant women in Africa will experience anaemia [2]. Iron deficiency accounts for 50–75% of these cases [3]. The common causes of pregnancy-related anaemia in Africa include nutritional disorders, malaria, infections, haemoglobinopathies, haemorrhage, and chronic diseases [4]. The condition is associated with an increased risk of morbidity and mortality for the mother and the baby during pregnancy and postpartum. Common complications related to anaemia in pregnant and postpartum women include low birthweight, preterm birth, postpartum haemorrhage, maternal depression and other psychosocial events, stillbirth, and neonatal mortality [5, 6].

The World Health Organization (WHO) recommends Hb testing at each trimester of pregnancy as a proxy screening for iron deficiency anaemia [7]. After delivery, Hb levels should be measured within 24 to 48 h in women with blood loss above 500 mL, women with uncorrected anaemia diagnosed within antepartum, or women exhibiting signs and symptoms suggestive of anaemia postnatally [7, 8]. The WHO classifies anaemia during pregnancy and puerperium (the first 6 weeks after childbirth during which the mother’s reproductive organs return to their original nonpregnant state) into three categories of severity: (1) mild anaemia (Hb levels 9 to 10.9 g/dL), (2) moderate anaemia (Hb levels 7.0 to 8.9 g/dL), and (3) severe anaemia (Hb levels less than 7.0 g/dL) [9].

Current WHO guidelines recommend that women diagnosed with obstetric anaemias should be treated with high-dose oral iron at 120 mg of elemental iron daily until Hb rises to normal (Hb 11.0 g/dL or higher) [7]. However, oral iron, though cheap, usually takes several months of treatment to correct anaemia and replenish iron stores [10]. Also, about 70% of patients on oral iron (particularly ferrous sulphate) experience gastrointestinal adverse effects such as vomiting, constipation, diarrhoea, and abdominal pain [10–12]. These adverse effects are often due to unabsorbed iron and ultimately limit treatment adherence, further posing a challenge to achieving optimum correction of anaemia by the end of the puerperium. As an alternative, some intravenous iron preparations can be given as a single dose and are suitable for patients who respond poorly to oral iron and moderately anaemic women that require more rapid iron replacement for symptom control [8]. Intravenous iron corrects anaemia faster and in fewer doses than oral iron, requiring less patient-provider interaction [8]. This situation is ideal in LMICs where the loss to follow-up is common for various reasons, especially in the postpartum period [13, 14]. Intravenous iron administration presents an opportunity to rapidly treat moderate and severe anaemia during pregnancy, thereby minimising the risk of poor maternal, foetal, and newborn outcomes.

Nigeria accounts for about 34% of global maternal deaths [15] and has a high prevalence of iron deficiency anaemia among pregnant women ranging from 25 to 45.6% [16]. Whereas clinicians know of intravenous iron, its use is not widespread for treating pregnancy-related iron deficiency anaemia [17]. This may be due to common misconceptions about the treatment modalities for iron deficiency anaemia, which have been reported in similar settings to Nigeria [10]. One of these misconceptions is that oral iron is always effective if patients tolerate high daily doses. However, the recommended thrice daily dosing to treat mild to moderate anaemia in pregnancy is associated with poor adherence [18, 19], with compliance rates in Nigeria estimated to be as low as 65% [19]. Another common misconception is that intravenous iron is associated with a high risk of anaphylaxis and may increase the risks of infection and oxidative stress and should be restricted to severe cases of anaemia [10]. Contrary to this assumption, evidence suggests a shallow risk of life-threatening allergic reactions with all intravenous iron formulations [10]. Furthermore, data from meta-analyses and large observational studies show that, in comparison with oral iron or no iron, short-term intravenous iron therapy does not present an increased risk of infection [10, 20, 21].

Newer intravenous (IV) iron formulations, such as ferric carboxymaltose (FCM), which have fewer adverse effects than high-molecular weight iron dextran and has been demonstrated to be better tolerated than many other intravenous iron preparations, as well as oral iron, are now available [22]. A single high dose of FCM can be infused in 15 min, rapidly replenishing iron stores [23]. FCM has also been demonstrated to be cost-effective in correcting anaemia in comparison with the use of red blood cell transfusion [24], IV iron sucrose [25, 26], and ferric derisomaltose [26]. One study in the UK concludes that FCM is likely to be the least costly and most effective IV iron therapy available [26].

FCM has potential promise in treating obstetric anaemias in Nigeria and many other LMICs. In Nigeria, pregnant women seek antenatal care relatively late, with most first-time visits in the middle to the late second trimester, and less than 60% attend the recommended four or more antenatal visits [27]. The implication is that the detection of anaemia is also often late. Thus, FCM may be more effective than oral iron in Nigeria, even though the formulation is not yet on the essential drug list [28]. The IV versus oral iron clinical trials are ongoing to test this hypothesis in pregnancy (IVON trial) [12] and postpartum (IVON-PP trial ISRCTN51426226). This proposed implementation science research (IVON-IS) seeks to complement the IVON and IVON-PP trials by using continuous quality improvement and participatory action research to evolve and test strategies to strengthen existing diagnostic processes for anaemia in pregnancy and postpartum. The study will also test strategies to strengthen referral pathways and facilitate the routine treatment of moderate to severe anaemia with FCM within Nigeria’s health system.

Specifically, the objectives of the study are to:

- Identify opportunities and strategies to routinise and strengthen screening for anaemia to identify and target pregnant and postpartum women who stand to benefit from intravenous FCM administration
- Identify health system capacity needs at different levels required for FCM administration during pregnancy and options for immediate postpartum administration
- Identify potential care and referral pathways and understand the barriers and facilitators to uptake and user experiences
- Develop, test, and iterate strategies for improving the screening process for anaemia and FCM administration within the existing health system.

## Methods

**Fig. 1:**
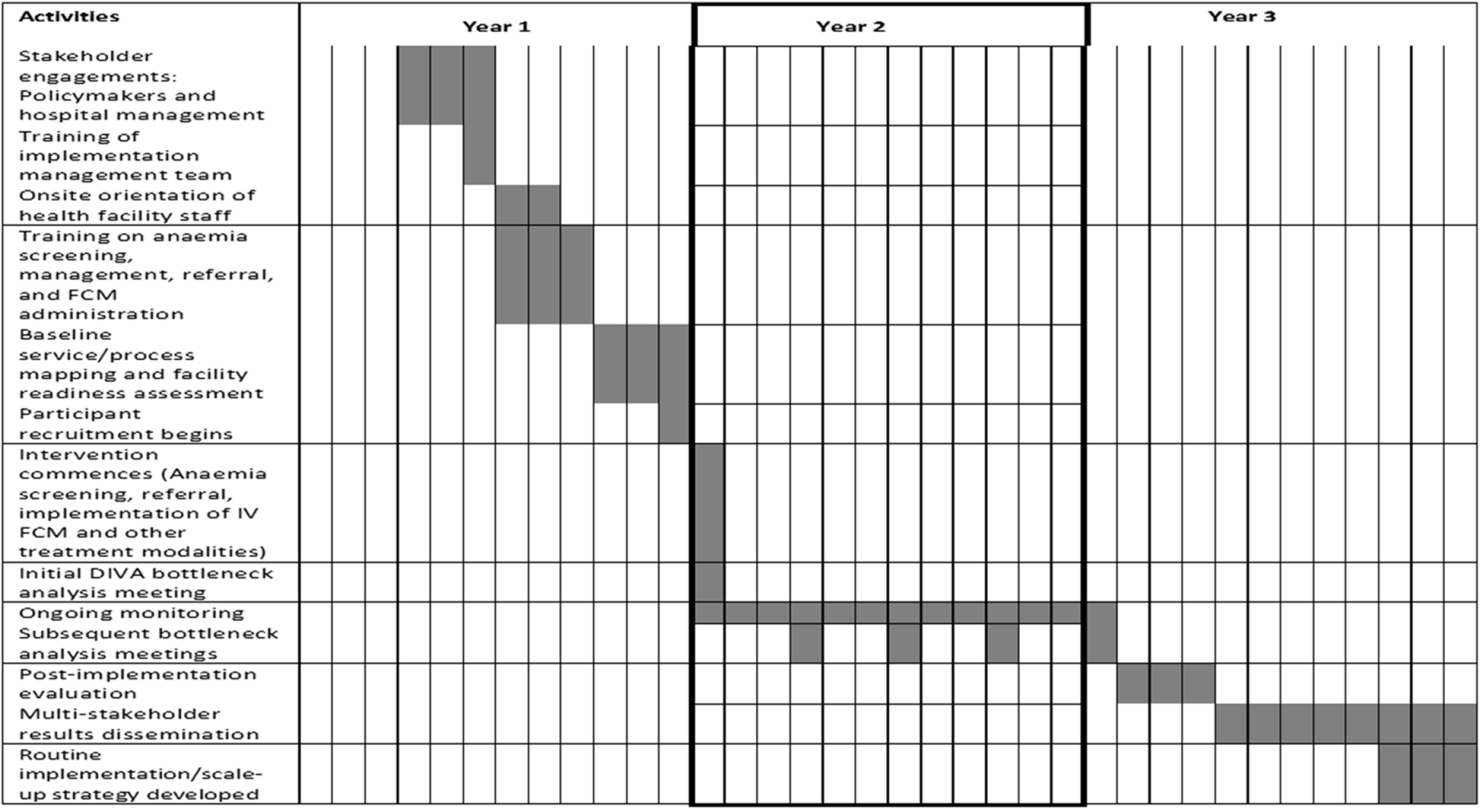
Proposed 36-month study timeline

The study adheres to the Standards for Reporting Implementation Studies (StaRI) statement and checklists from the EQUATOR network [29, 30] (see Additional file 1). The timelines for the study are presented in Fig. 1.

### Study setting

IVON-IS will be conducted in Lagos State, South West, Nigeria. Specifically, implementation outcomes will be tested in a cluster of six health facilities that have not yet been exposed to training or implementation related to the ongoing IVON trials. The facilities span public (government-owned) and private (individual/organisation-owed) sectors in the state. The cluster will include one tertiary hospital, two secondary hospitals, one comprehensive primary health centre (PHC) in the public sector, and two private hospitals with varied patient bases. The selected facilities will be in proximity and linked to the state’s existing referral process. The cluster will represent a sub-unit of the health system and reflect the points of antenatal and postnatal care for pregnant women. Indeed, in Lagos, public health facilities manage 27% of deliveries in the state, while private health facilities take up about 48% [27]. Using projected data from our ongoing IVON clinical trials, we estimate that we will screen about 4000 pregnant and postpartum women per annum and have approximately 400 women eligible for treatment for anaemia.

### Study interventions and implementation processes

**Fig. 2:**
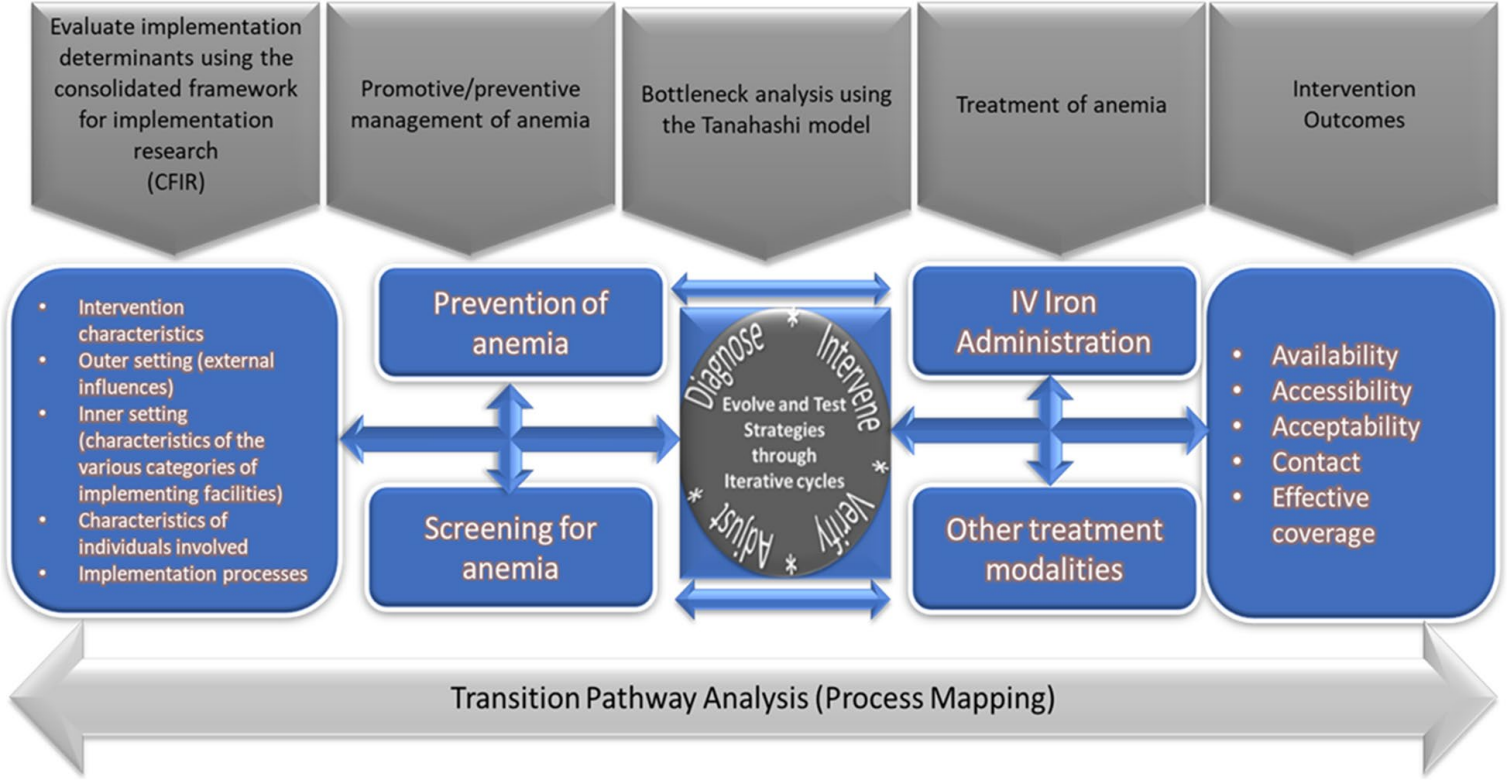
Scope of the IVON implementation research

The IVON-IS will aim to test the routine implementation of two interventions along the management pathway for anaemia in pregnancy and postpartum (i.e. screening for anaemia and treating anaemia using FCM). The study will also examine the linkages and interactions of patients, health system actors, and processes within and between health facilities as women transition through the health system (Fig. 2).

### Implementation strategy

**Fig. 3:**
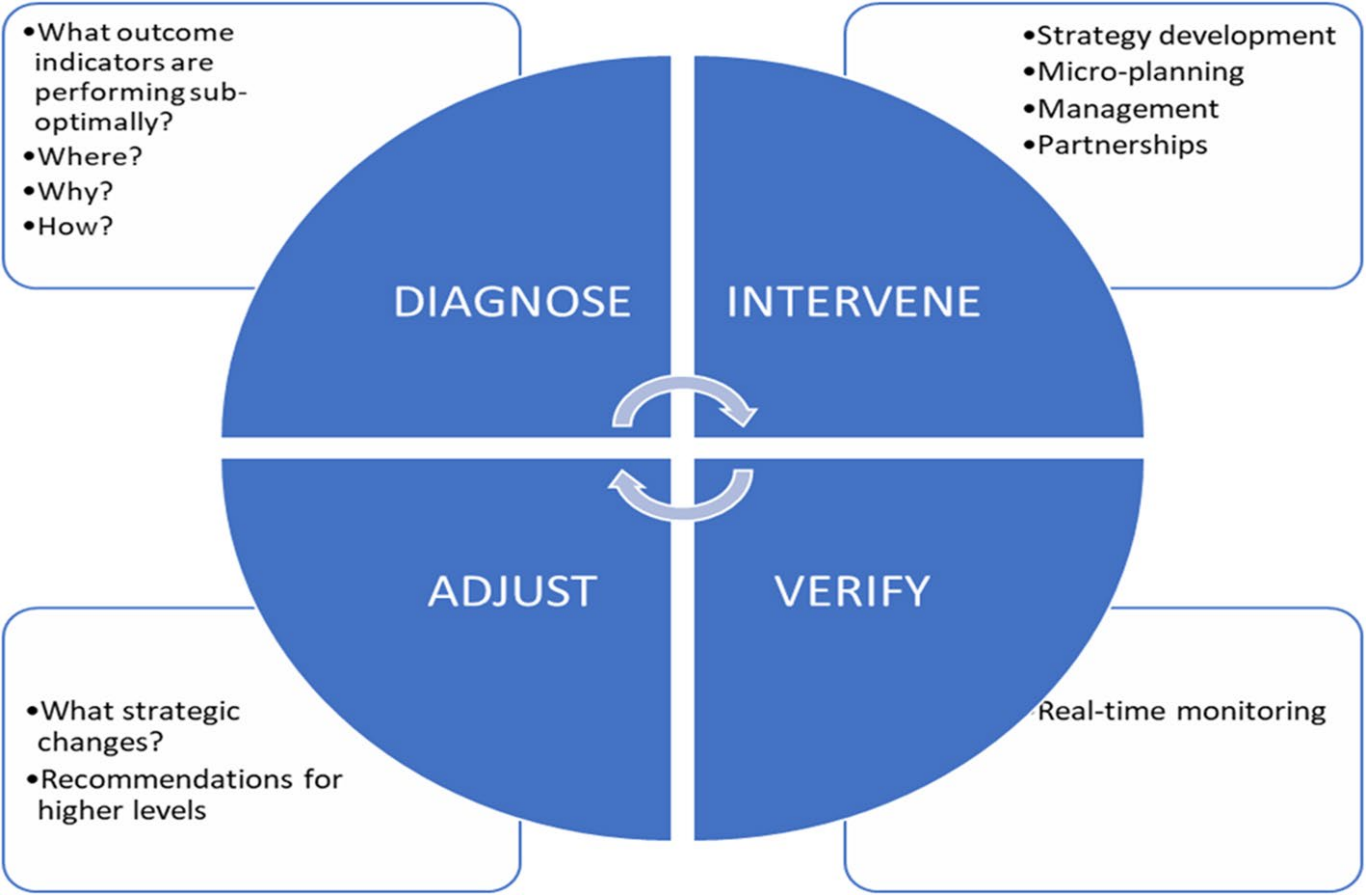
The DIVA Cycle

Continuous quality improvement, which incorporates the Tanahashi model for health system evaluation and a variant of the Plan-Do-Study-Act (PDSA) cycle known as the Diagnose-Intervene-Verify-Adjust (DIVA) model will be employed to identify and improve implementation bottlenecks using participatory approaches (Fig. 3). Tanahashi’s model for health service coverage emphasises the quality and effectiveness when attaining an equitable and effective coverage [31, 32]. DIVA has been successfully applied to identify and improve bottlenecks in decentralised health systems across sub-Saharan Africa, including Nigeria [31, 33, 34]. For our study, DIVA will be implemented in quarterly cycles.

An implementation management team (IMT) comprising health system actors (health care providers, health services managers, policymakers), health services users/interest groups (patients, families, community/ward development committees), and academic researchers will be convened. We will commence with an introductory meeting with the IMT. This will be followed by a pre-implementation training of the IMT on the bottleneck analysis approach and tools. The bottleneck analysis approach identifies barriers to effective coverage of healthcare services and develops strategies for improvement. Bottleneck analysis workshops will be conducted quarterly during the implementation of the project. The IMT will undertake activities to:

1. Understand and document what anaemia screening and treatment currently look like and what the patient journeys are, considering the levels of the health system and facility and staffing requirements across the care cascade (e.g. testing, referral, treatment, follow-up)
2. Identify elements that need to be strengthened and levels at which new strategies for screening, referral, and various anaemia treatment modalities, such as iron-folic acid and intermittent preventive treatment of malaria during pregnancy, can be introduced
3. Co-design new strategies with key stakeholders, including government officials, along with an implementation framework that articulates key implementation-related questions
4. Test approaches for implementing co-developed context-sensitive strategies using pre-designed evaluation methods for comparison and iteration
5. Understand and document user experiences, as well as key barriers and facilitators to anaemia screening and testing

#### DIVA processes and outcomes

**Fig. 4:**
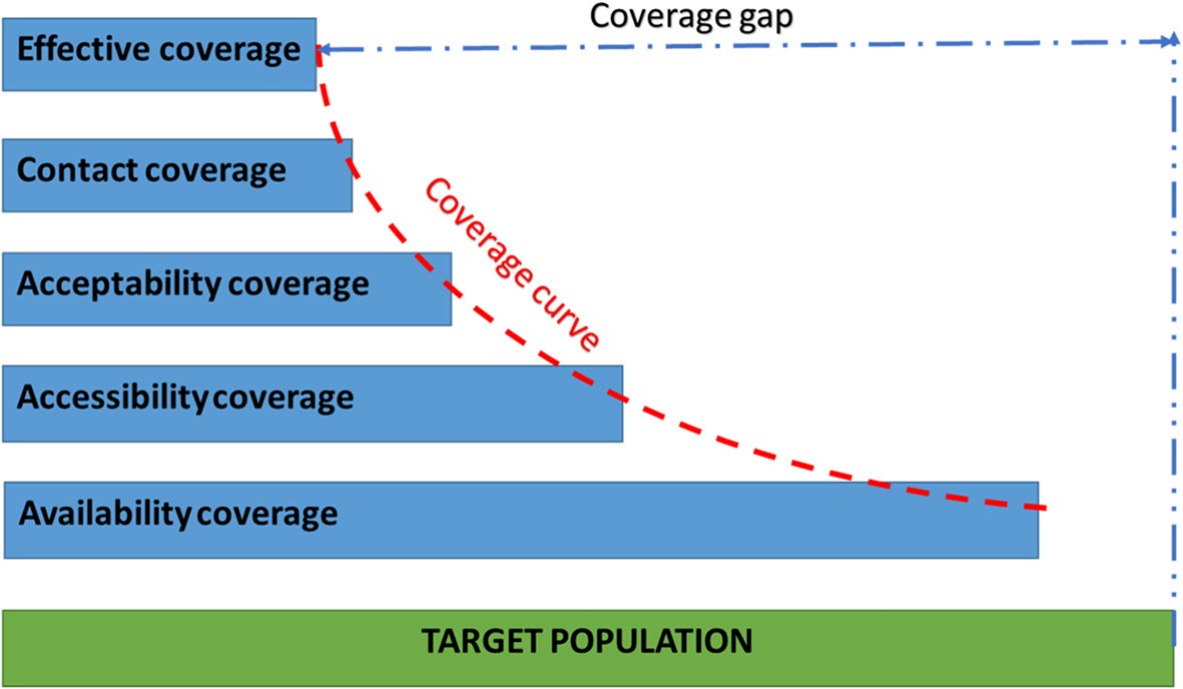
Adapted from the Tanahashi model for health system evaluation

The outcomes of interest will be informed by Tanahashi’s model for health system evaluation [32]. Tanahashi argued that health care coverage should not measure the percentage number of people reached but rather measure the percentage of the number of people who received quality service. He then proposed a model to identify and address the barriers to improving effective coverage of interventions. The Tanahashi model consists of five domains (Fig. 4):

1. Availability coverage: availability of human resources and essential commodities
2. Accessibility coverage: accessibility of distribution points for interventions
3. Acceptability coverage: proportion of the population willing to use the service
4. Contact coverage: proportion of the target population who use the service
5. Effective/quality coverage: proportion of the target population who received quality and/or satisfactory service

During the ‘diagnose’ phase of DIVA, the IMT will conduct bottleneck analyses across the Tanahashi model domains to identify constraints to effective coverage of the interventions (preventive, diagnostic, and management modalities). The difference between target coverage and observed coverage for each domain indicator will be identified as a measure of the bottleneck.

**Fig. 5:**
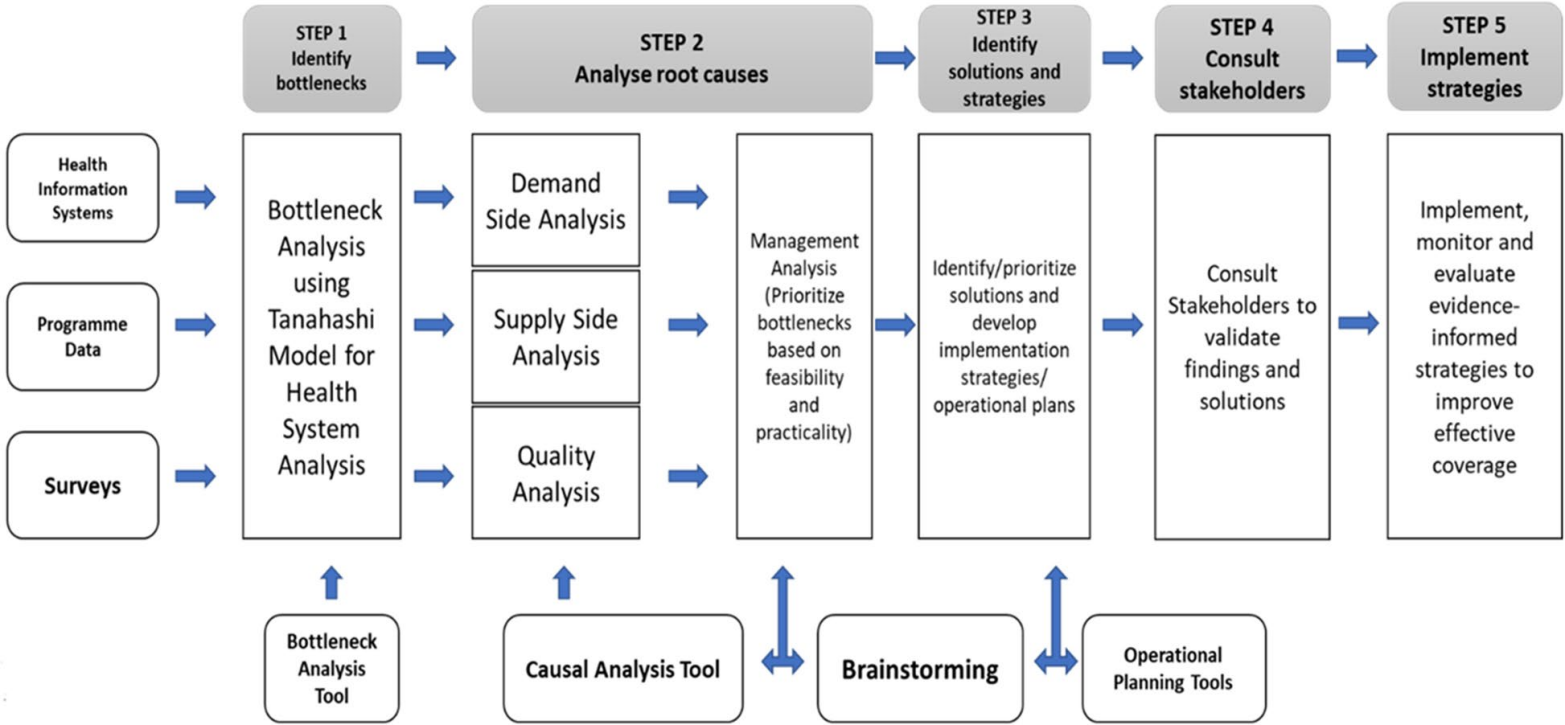
The process framework for bottleneck identification, analysis, and improvement

Using techniques and tools like brainstorming, the ‘5 whys’ technique, affinity, and driver diagrams, we will guide the IMT to identify immediate, proximal, and distal causes of identified bottlenecks. This step is known as a root cause/causal analysis. In the ‘intervene’ phase, the IMT will brainstorm plausible solutions and strategies to address these bottlenecks. Subsequently, proffered strategies will be converted into action plans along with specific quality/coverage targets for the next implementation quarter. During the ‘verify’ phase, the implementation of planned activities will be monitored through existing supportive supervision, monitoring, and evaluation mechanisms year-round. This will help the early detection of deviation or lag while also optimising the implementation fidelity of planned activities. At any point during verification, implementation challenges identified will be addressed to ensure strategies are carried out as planned and are on track towards attaining targets within stipulated time frames. This forms the ‘adjust’ phase [33, 34] (Fig. 5).

#### DIVA outcome measures

Table 1 highlights the study indicators to be quantitatively monitored and improved. The primary outcome of the DIVA process will be the improvement in the implementation fidelity (effective coverage) to the three management processes along the continuum of care as depicted in the scope (Fig. 2). These are:

- Process of screening for anaemia in pregnancy and postpartum
- Support along the care and referral pathway following diagnosis of anaemia in pregnancy and postpartum
- Procedural guide for the infusion of IV ferric carboxymaltose (as deployed in the IVON trial)

**Table 1:** Outcome measures and indicators to be monitored and improved

Tanahashi model domain	IVON-IS outcome measure	Operational definition		
		Preventive/promotive care	Existing management procedures	FCM treatment
Availability coverage	Trained human resource availability	The proportion of target health workers at each site who have been trained on the screening and other preventive care protocols for antepartum and postpartum anaemia (including health education and nutrition)	The proportion of target health workers at each site who have been trained on the screening and management protocols for antepartum and postpartum anaemia (including preventive, promotive, diagnostic, and treatment)	The proportion of target health workers at each site who have been trained in administering FCM. These health workers are from health facilities that have not yet been exposed to training or implementation of IV FCM.
Accessibility coverage	Service accessibility	The proportion of women attending antenatal and postnatal clinics who were offered screening and promotive care	The proportion of women diagnosed with anaemia who were prescribed treatment for anaemia	The proportion of women eligible to receive the IV FCM for pregnancy and/or postpartum anaemia who were offered the infusion.
Acceptability coverage	Acceptability	The proportion of eligible women who were screened and who utilised other promotive care provided	The proportion of eligible women who used the prescribed treatment option	The proportion of women eligible to receive the IV FCM for pregnancy and/or postpartum anaemia who consented to receive the infusion
Contact coverage	Service utilisation	Same with acceptability	Same with acceptability	The proportion of women eligible to receive the IV FCM for pregnancy and/or postpartum anaemia who were administered the infusion irrespective of the protocols.
Effective/quality coverage^a^	Implementation fidelity	The proportion of eligible women screened for anaemia who adhered to the post-screening plan (e.g. referral for treatment of diagnosed anaemia or prescribed nutritional supplements)	The proportion of women with anaemia who used the treatment option according to the prescribed plan (e.g. dose/duration)	The proportion of women eligible to receive the IV FCM for pregnancy and/or postpartum anaemia who were administered the infusion according to the protocols.

^a^Primary outcome of DIVA

### Implementation evaluation

A multi-methods approach will be employed in the evaluation. Methods will involve quantitative and qualitative approaches embedded within continuous quality improvement. The evaluation will consist of formative, process, and outcome evaluations.

#### Formative evaluation

Formative (baseline) evaluation will include surveys of Organisational Readiness to Implement Change [35], Acceptability of Intervention Measure, Intervention Appropriateness Measure, and Feasibility of Intervention Measure [36], as well as a descriptive analysis of the indicators of the Tanahashi constructs listed in Table 1.

Guided by the Consolidated Framework for Implementation Research (CFIR) [37], the determinants (barriers and facilitators) of implementing routine screening and management of anaemia during pregnancy and postpartum will be evaluated using qualitative methods (Additional file 2). Furthermore, opportunities and strategies to improve implementation outcomes will be evolved using DIVA’s participatory approaches and focus group discussions (FGDs) involving implementers, patient representatives, policymakers, and researchers. We anticipate conducting 12 FGDs, each involving 8–12 participants. Key informant interviews with doctors, nurses, and other health workers (e.g. antenatal care, delivery, and postpartum officers at all health facility levels) will be conducted on a rolling basis until data saturation is achieved (a point in data collection when no additional issues or insights are identified from interviewing additional participants).

#### Process evaluation

Process evaluation will be conducted using the DIVA approach with the aid of the monitoring indicators described earlier. Furthermore, a process map will be developed as an output to communicate the understanding of the flow of patients as they transition across various facilities and levels of the health system to receive care from antenatal to postnatal periods and from screening for anaemia through the referral process to receiving various treatment options, including nutrition, medication, and IV FCM. The process map will also provide information about feasible services that can be delivered effectively across various categories of health facilities.

#### Outcome (summative) evaluations

Outcome evaluations will be conducted at the end line of the study. The primary outcome of the impact evaluation will be the effective coverage measures (implementation fidelity) indicated in Table 1. The secondary outcomes will be a repeat of the surveys carried out at baseline to evaluate the change in measures. Patient satisfaction with the interventions will also be surveyed.

Qualitative summative evaluation will be conducted through interviews and FGDs with the same stakeholder groups listed under formative evaluation. The normalisation process theory (NPT) [38] will guide the interviews and discussions, which will seek to understand how IV FCM was implemented, embedded, and integrated into the health system for the routine management of anaemia in pregnancy and postpartum.

#### Data analysis

Descriptive analysis using frequencies and percentages will be performed on categorical variables, while a Shapiro–Wilk test of normality will be performed on continuous variables. Variables with a normal distribution (non-significant (*p* > 0.05)) will be presented as means with standard deviations, while non-normally distributed data will be presented as medians with interquartile ranges. Univariable and multivariable ordinal and binary logistic regression will be used to evaluate the determinants of categorical implementation outcomes. Simple and multiple linear regression analyses will assess the changes in continuous variables.

Qualitative data recorded from interviews and DIVA will be transcribed verbatim. Familiarisation with the audio recording and reflexive notes recorded during transcription will be carried out before the analysis. Transcribed data will be coded deductively (guided by the NPT and CFIR) [37, 38] and inductively to generate themes emerging from the data. We will use thematic and framework analysis to capture the underlying concepts and patterns.

## Discussion

The IVON-IS is a novel study in Africa. Complementary to the IVON and IVON-PP clinical trials, the IVON-IS seeks to routinise the use of an IV iron formulation by overcoming contextual and systemic barriers using continuous quality improvement.

While misconceptions around the use of IV iron formulations are global, systemic constraints to use may be contextual [10]. Thus, this study provides an opportunity to contribute to both contextual and generalisable knowledge on the adoption and administration of FCM in the management of obstetric cases of iron deficiency anaemia. The study not only focuses on the endpoint but also seeks to improve the continuum of care related to obstetric anaemia. Routine nutrition prescriptions and practices, health promotion, screening for anaemia, and referral processes will also be strengthened.

The study will throw light on health system constraints to address the highly prevalent problem of obstetric anaemia in Nigeria, which contributes to the high maternal and infant morbidity and mortality. Furthermore, the study is expected to evolve transferable knowledge on addressing these bottlenecks within context. Thus, lessons from this IVON-IS study will inform scale-up across Nigeria and the adoption of the interventions and strategies across other African countries.

The methods and strategies used in this study will contribute to the body of knowledge in both implementation and improvement sciences applicable to LMICs. The Deming Cycle or PDSA is considered an intervention in improvement science but a method or strategy in implementation science [39, 40]. Thus, the PDSA is viewed as an intersection between both disciplines [40]. This four-step iterative technique is often used to solve problems and improve organisational processes [41]. In contrast, however, the DIVA model used in the IVON-IS study, though an adaptation of the PDSA, is designed to improve the implementation of interventions within complex health systems common in LMICs [39]. Incorporating the Tanahashi health systems evaluation model into DIVA provides an opportunity to apply systems thinking in quality improvement. This approach also strengthens the decentralised evolution of change strategies towards adoption and optimal implementation of new interventions within existing health system structures and processes. It is hoped that lessons learned from the methodological approach used in the IVON-IS will contribute to building capacity and advancing the application of implementation science to strengthen health systems in Africa and beyond.

## Supplementary Information

**Additional file 1.** StaRI Checklist.

**Additional file 2.** Constructs of the CFIR as adapted for the IVON-IS project.

## Data Availability

Not applicable.

## Abbreviations

CFIR: Consolidated Framework for Implementation Research
DIVA: Diagnose-Intervene-Verify-Adjust
FCM: Ferric carboxymaltose
FGD: Focus group discussion
Hb: Haemoglobin
IMT: Implementation Management Team
IVON: Intravenous Versus Oral Iron-Antepartum Trial
IVON-IS: Intravenous Versus Oral Iron-Implementation Study
IVON-PP: Intravenous Versus Oral Iron-Postpartum Trial
LMICs: Low- and middle-income countries
NPT: Normalisation process theory
PDSA: Plan-Do-Study-Act
