# Supplementary material for: A continuous quality improvement strategy to strengthen screening practices and facilitate the routine use of intravenous iron for treating anaemia in pregnant and postpartum women in Nigeria: a study protocol
Source: Implement Sci Commun. 2023 Mar 7;4:22. doi: 10.1186/s43058-023-00400-y (PMC9993807; doi:10.1186/s43058-023-00400-y)
Supplement: Supplementary file 2 — Additional file 2. Constructs of the CFIR as adapted for the IVON-IS project. [file 43058_2023_400_MOESM2_ESM.docx]

**Additional File 2:** Constructs of the CFIR as adapted for the IVON-IS project

| **Construct** | **Short description of the construct** | **IVON-IS adaptation** |
| --- | --- | --- |
| **I. Intervention characteristics** | |  |
| Intervention source | Perception of key stakeholders about whether the intervention is externally or internally developed. | Do stakeholders perceive the IVON intervention as being developed internally or externally? |
| Evidence strength and quality | Stakeholders’ perceptions of the quality and validity of evidence supporting the belief that the intervention will have desired outcomes. | How do stakeholders perceive the evidence supporting the IVON intervention and its desired outcomes? |
| Relative advantage | Stakeholders’ perception of the advantage of implementing the intervention versus an alternative solution. | Do stakeholders perceive/believe that the IVON intervention worked better than other alternative solutions? |
| Adaptability | The degree to which an intervention can be adapted, tailored, refined, or reinvented to meet local needs. | Can the IVON intervention be tailored, refined, or reinvented for local contexts/needs? |
| Trialability | The ability to test the intervention on a small scale in the organisation and to reverse course (undo implementation) if warranted. | Can the IVON intervention be tested on a smaller scale within the health facility, and can it be reversed if needed? |
| Complexity | Perceived difficulty of implementation, reflected by duration, scope, radicalness, disruptiveness, centrality, intricacy, and number of steps required to implement. | How difficult do stakeholders perceive the IVON intervention to be? |
| Design quality and packaging | Perceived excellence in how the intervention is bundled, presented, and assembled. | How do stakeholders perceive the design and presentation of the IVON-IS? |
| **II. Outer setting** |  |  |
| Patient needs and resources | The extent to which patient needs, as well as barriers and facilitators to meet those needs, are accurately known and prioritized by the health facility. | Are patients’ needs known and prioritized by the health facility, as well as barriers and facilitators that affect meeting those needs? |
| External policy and incentives | A broad construct includes external strategies to spread interventions, including policy and regulations (governmental or other central entity), external mandates, recommendations and guidelines, pay-for-performance, collaboratives, and public or benchmark reporting. | Are there any policies, recommendations, or mandates to implement the IVON intervention? |
| **III. Inner setting** |  |  |
| Structural characteristics | The size and type of health facility within the Nigerian health system. | How old and big is the health facility? What are the health facility type and levels within the Nigerian health system? |
| Networks & Communications | The nature and quality of webs of social networks and the nature and quality of formal and informal communications within an organization. This includes the referral processes within and between facilities. | How are social networks organized within the health facility? Is there good communication between individuals, units, services, and organization levels? |
| Culture | Norms, values, and basic assumptions of a given health facility. | What are the health facility's cultural norms, values, and basic assumptions? |
| Implementation climate | The absorptive capacity for change, shared receptivity of involved individuals to an intervention, and the extent to which use of that intervention will be rewarded, supported, and expected within their facility. | How receptive are healthcare workers/staff in the institutions to the IVON intervention? Will the use of the IVON intervention be rewarded and supported by leaders? |
| Tension for Change | The degree to which stakeholders perceive the current situation as intolerable or needing change. | Do stakeholders perceive the current situation as needing change? |
| Compatibility | The degree of tangible fit between meaning and values attached to the intervention by involved individuals, how those align with individuals’ norms, values, and perceived risks and needs, and how the intervention fits with existing workflows and systems. | How well does the intervention fit with the organizational culture? Do stakeholders perceive a “match” between the meaning they attach to the IVON intervention, and the meaning attached to it by upper management? |
| Relative Priority | Individuals’ shared perception of the importance of the implementation within the organization. | How important is the IVON intervention when compared to the competing priorities of individual stakeholders, upper management, and the health facility? |
| Organizational Incentives & Rewards | Extrinsic incentives, such as goal-sharing awards, performance reviews, promotions, and raises in salary, and less tangible incentives, such as increased stature or respect. | Are there tangible incentives to adopt the IVON intervention, such as promotions, salary raises, improved autonomy, or other forms of recognition? |
| Goals and Feedback | The degree to which goals are communicated, acted upon, and fed back to staff, and alignment of that feedback with goals. | Are the health facility goals communicated to staff, and are they given feedback about progress ad performance? |
| Learning Climate | A climate in which: a) leaders express their fallibility and need for team members’ assistance and input; b) team members feel that they are essential, valued, and knowledgeable partners in the change process; c) individuals feel psychologically safe to try new methods; and d) there is sufficient time and space for reflective thinking and evaluation. | Do leaders openly share their own mistakes and needs? Do team members feel essential and valued in the change process? Do individuals feel safe trying new methods? Is there enough time and space for reflective thinking and evaluation? |
| Readiness for Implementation | Tangible and immediate indicators of organizational commitment to its decision to implement an intervention. | Are leaders and managers committed to the decision to implement the IVON intervention? |
| Leadership Engagement | Commitment, involvement, and accountability of leaders and managers with the implementation. | Are leaders and managers committed and involved in the IVON implementation? |
| Available Resources | The resources dedicated to the implementation and ongoing operations include money, training, education, physical space, and time. | Have the necessary resources (money, time, personnel) been defined, and are they available? |
| Access to Knowledge & Information | Ease of access to digestible information and knowledge about the intervention and how to incorporate it into work tasks. | Is the information about the IVON intervention easily accessible and incorporated into work tasks? |
| **IV. Characteristics of stakeholders involved** | | |
| Knowledge and beliefs about the intervention | Individuals’ attitudes toward and the value placed on the intervention, as well as familiarity with facts, truths, and principles related to the intervention. | What are health workers, patients, and policymakers’ attitudes, values, and familiarity with facts associated with the intervention? |
| Self-efficacy | Individuals believe in their capabilities to execute courses of action to achieve implementation goals. | How much do health workers believe in their capabilities to execute the IVON intervention? |
| An individual stage of change | Characterization of an individual's phase as they progress toward skilled, enthusiastic, and sustained use of the intervention. | Are health workers progressing toward skilled, enthusiastic, and sustained use of the IVON intervention? |
| Individual identification with the organisation | A broad construct related to how individuals perceive the organisation, their relationship, and their degree of commitment to that organization. | How do stakeholders from various stakeholder groups perceive the health facility? What is their relationship to the health facility, and how committed are they? |
| Other personal attributes | A broad construct to include other personality traits such as tolerance of ambiguity, intellectual ability, motivation, values, competence, capacity, and learning style. | What other personal traits of stakeholders may impact the IVON implementation success (i.e., tolerance of ambiguity, intellectual ability, motivation, values, competence, capacity, and learning style)? |
| **V. Process** | | |
| Planning | The degree to which a scheme or method of behaviour and tasks for implementing an intervention are developed in advance and the quality of those schemes or methods. | Have the proper steps to promote effective implementation been established and put in place? |
| Engaging | We are attracting and involving appropriate individuals in the implementation and use of the intervention through a combined strategy of social marketing, education, role modelling, training, and other similar activities. | Who is involved in the implementation? Does the implementation effort have a champion, and how were they brought in? What is their role in the health facility and in the implementation? |
| Opinion Leaders | Individuals in an organization who have a formal or informal influence on the attitudes and beliefs of their colleagues concerning implementing the intervention. | Who are the expert or peer opinion leaders who influence others? |
| Formally Appointed Internal Implementation Leaders | Individuals from within the organization who have been formally appointed with responsibility for implementing an intervention as coordinator, project manager, team leader, or other similar roles. | Who are the individuals who work for the health facility responsible for implementing the intervention as coordinators, project managers, team leaders, etc.? |
| Champions | “Individuals who dedicate themselves to supporting, marketing, and ‘driving through’ an [implementation]”, overcoming indifference or resistance that the intervention may provoke in an organization. | Who are the people who actively and enthusiastically promote the implementation? |
| External Change Agents | Individuals who are affiliated with an outside entity who formally influence or facilitate intervention decisions in a desirable direction. | Who are the individuals external to the implementation process who facilitate intervention decisions (such as a knowledge broker)? |
| Executing | Carrying out or accomplishing the implementation according to plan. | Who are the people carrying out or accomplishing the implementation according to the plan? |
| Reflecting & evaluating | Quantitative and qualitative feedback about the progress and quality of implementation accompanied by regular personal and team debriefing about progress and experience. | What is the feedback about the progress and quality of implementation, including regular personal and team debriefing about progress and experience? |
